# Comparison of Adductor Canal Block and Femoral Nerve Block for Early Ambulation After Primary Total Knee Arthroplasty: A Randomized Controlled Trial

**DOI:** 10.7759/cureus.6331

**Published:** 2019-12-09

**Authors:** Promil Kukreja, Cara Bevinetto, Brandon Brooks, Haley McKissack, Tyler P Montgomery, Bradley Alexander, Ashish Shah

**Affiliations:** 1 Anesthesiology, University of Alabama School of Medicine, Birmingham, USA; 2 Anesthesiology and Preoperative Medicine, University of Alabama School of Medicine, Birmingham, USA; 3 Anesthesiology and Perioperative Medicine, University of Alabama School of Medicine, Birmingham, USA; 4 Orthopaedics, University of Alabama School of Medicine, Birmingham, USA

**Keywords:** adductor canal block, femoral nerve block, total knee arthroplasty, regional anesthesia

## Abstract

Purpose: The purpose of this study was to assess the efficacy of adductor canal block (ACB) as compared to femoral nerve block (FNB) in ambulation distance, opioid consumption, and physical therapy participation on postoperative days (PODs) 1 and 2 after total knee arthroplasty (TKA). We hypothesized ACB would have increased the ambulation distance and decreased the opioid consumption in comparison to FNB.

Methods: All elective TKAs at a single institution, age 18 and older, without existing neurologic or anatomic deficit in the operative limb, were considered. Participants were randomized 1:1 to receive either an ACB (AC group) or a FNB (FN group), in addition to standard care. Visual analog pain scores (VAS) and oral morphine equivalents (OMEs) were recorded preoperatively, in post-anesthesia care unit (PACU), and on PODs 1 and 2. Postoperative ambulation distance was recorded on PODs 1 and 2. Patient satisfaction with analgesia and physical therapist-rated participation in therapy sessions was obtained as well.

Results: From 2014 to 2015, 84 participants were recruited: 41 in FN, and 43 in AC. On POD 1, mean ambulation distances in AC and FN were 70.2 and 48.5 ft, respectively (p = 0.045). On POD 2, mean ambulation distances in AC and FN were 129.0 and 106.4 ft, respectively (p = 0.225). VAS, OME, satisfaction, and physical therapy participation were not significantly different.

Conclusions: Ambulation after TKA is superior with ACB on the first POD, but there is no difference in VAS scores, OME, patient satisfaction, or ambulation on POD 2.

## Introduction

Peripheral blockade of the femoral nerve (FN) has long been used for perioperative pain control in total knee arthroplasty (TKA). These blocks provide effective analgesia, which is reflected in patient satisfaction with surgical pain relief. However, one of the main drawbacks of the femoral nerve block (FNB) is a dense motor block of the quadriceps muscle, which can delay aggressive physical therapy and subsequent recovery from surgery [1]. Recently, there has been an increasing interest in performing a more targeted, distal block that avoids the motor blockade of the FNB. One of the proposed blocks is the adductor canal block (ACB), which is thought to avoid the quadriceps motor blockade while providing noninferior analgesia compared to FNB [1-2]. Current investigative reports have only provided preliminary data, leaving questions about ACB as compared to FNB unanswered with respect to ambulation postsurgery.

 The FNB catheters have been used to provide analgesia for lower extremity procedures for many years. The catheter is placed near the FN and a continuous infusion provides a dense nerve block in the distribution of the FN. This includes blockade of the anterior and posterior divisions supplying the middle cutaneous, medial cutaneous, and muscular (sartorius) branches in the anterior division and the saphenous, muscular (to quadriceps), and articular branches of the posterior division. In particular, the muscular branch supply to the quadriceps makes walking and participating in physical therapy difficult. 

 An ACB can be expected to include the saphenous nerve, vastus medialis, medial femoral cutaneous, articular branches from the obturator and the medial retinacular nerves. This distribution provides the innervation for the medial, anterior, and lateral portions of the knee. van der Wal et al. first demonstrated in 1993 the trans-sartorial approach to blocking the saphenous nerve [3]. In recent years, the ACB has been proposed as a potential successor to the FNB [1-2, 4-5]. Kwofie et al. investigated quadriceps strength and fall risk in volunteers finding that ACB significantly preserved motor strength and balance [5]. ACB also demonstrated superior analgesia compared to parenteral opioids alone. Further, Kim et al. performed a prospective, randomized, controlled trial comparing ACB to FNB with the end point of quadriceps strength, reported analgesia, and opioid intake showing that ACB provided similar analgesia but with less motor impairment [4]. However, no work to date has assessed ambulation distance in patients undergoing TKA on postoperative days (PODs) during their hospital stay.

 We hypothesize that the ACB will show improved postoperative ambulation distance as compared to FNB, while maintaining comparable analgesic effects. With better physical therapy participation and earlier ability to ambulate greater distances, we also hypothesize that there will be improved physical therapy participation, earlier discharge, and improved patient satisfaction.

## Materials and methods

**Figure 1:**
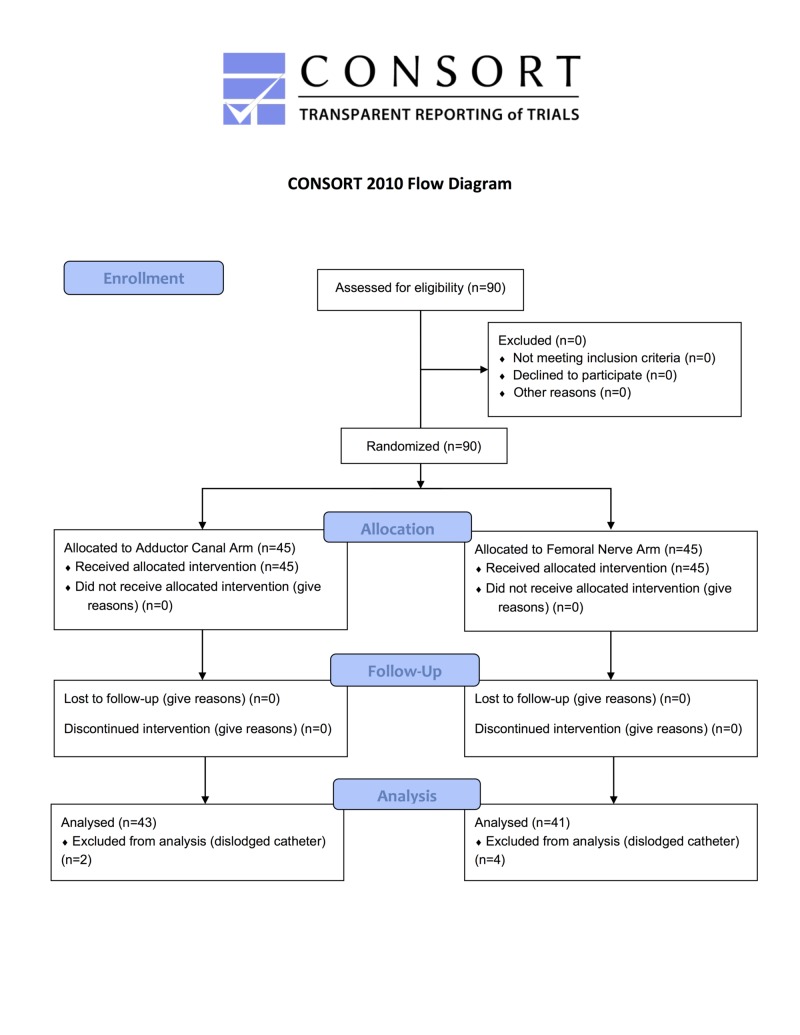
CONSORT flowchart of participant involvement.

This study was approved by the Institutional Review Board in accordance with the Declaration of Helsinki. All patients receiving an elective primary TKA at a single institution formed the population that study participants were recruited from (participant flowchart shown in Figure 1). Patients were considered eligible for the study if they were over 18 years of age at the time of surgery, planned to receive regional anesthesia in the course of the procedure, and were American Society of Anesthesiologists (ASA) Class I, II or III. Patients were excluded if there was no plan for regional anesthesia, if they had an allergy or prior adverse reaction to regional anesthesia, if any pre-existing neurologic or anatomic deficits were present in the limb to be operated on, or if a known history of a coagulopathy such as hemophilia or Von Willebrand’s disease was present.

Sample size was determined utilizing a binomial power calculator from www.sealedenvelope.com [6] for a superiority trial with a continuous variable. The authors agreed on a significance level of 5%, a cohort size which would achieve 80% power, and to use ambulation distance on the first POD as the outcome measure. Historically reported ambulation distances with different regional anesthesia after TKA [7] have reported a 50% difference on the first POD. Assuming this difference in our cohort, a sample size of 41 in each study arm was calculated as sufficient to achieve 80% power in assessing a significance level of 5%, assuming a 50% difference in ambulation distance on the first POD.

Once recruited, patients were randomized 1:1 to one of two treatment arms: adductor canal (AC) group (study group), or FN group (control group). The AC group received standard care and an ACB prior to TKA. The FN group received standard care and a FNB prior to TKA.

For block placement in the AC group, participants were placed in the supine position, with the limb to be blocked supinated 20 degrees to facilitate access to the anteromedial thigh. Standard noninvasive monitors were applied, and oxygen administered via nasal cannula. Parenteral midazolam and fentanyl were titrated to patient comfort. Standard skin sterilization, prepping, and draping were applied. Under ultrasound guidance, the needle was advanced into the adductor canal at mid-thigh level where, after a negative aspiration, a bolus of 20 mL 0.5% ropivacaine was injected in 5 mL aliquots under direct visualization. The catheter was then advanced 2-5 cm and secured to the skin with Tegaderm.

For block placement in the FN group, participants were placed in the supine position, with the inguinal area of the limb to be blocked exposed to facilitate access. Standard noninvasive monitors were applied, and oxygen administered via nasal cannula. Parenteral midazolam and fentanyl were titrated to patient comfort. Standard skin sterilization, prepping, and draping were applied. Under ultrasound guidance, the needle was advanced near the FN sheath and, after a negative aspiration, a bolus of 20 mL 0.5% ropivacaine was injected in 5 mL aliquots under direct visualization. The catheter was then advanced 2-5 cm and secured to the skin with Tegaderm. The local anesthetic infusion of 0.2% ropivacaine was continued postoperatively at the rate of 8 mL/h for first 48 h.

Both study groups received 1000 mg of acetaminophen, and 200 mg of celecoxib as preoperative multimodal regimen. Outside of the specific block, both study groups received the same care and data collection procedures during the preoperative, perioperative, and postoperative periods. Visual analog pain scores (VAS) were recorded in the immediate preoperative period, immediate postoperative period, 24 h, and 48 h. A standard pain control regimen was provided and total oral morphine equivalents (OMEs) was calculated for the immediate preoperative period, immediate postoperative period, 24 h, and 48 h. The total distance a participant was able to ambulate (in feet) was recorded on the first and second PODs. A participant’s overall satisfaction with their anesthesia was recorded, on a 0-10 scale, on the second POD. A participant’s physical therapist was surveyed for their level of participation in physical therapy sessions, on a 0-10 scale, on the second POD.

Statistical analyses were performed utilizing SAS statistical software. Means were calculated, and comparisons were made using a Student’s two-sample t-test with a 5% significance level.

## Results

This study was approved by our institution’s Institutional Review Board in accordance with the Declaration of Helinski. From 2014 to 2015, a total of 84 participants were included, with 43 participants recruited into the AC group and 41 into the FN group (Table 1). The average age of patients in the AC and FN groups were 63.4 and 65.4 years, respectively, and 53.5% of all patients (53.7% FN, 53.4% AC) were females. None of the aforementioned demographic characteristics were significantly different between groups. 

**Table 1:** Demographics of all subjects, ACB group, and FNB group. BMI, body mass index; ACB, adductor canal block; FNB, femoral nerve block

	Adductor canal	Femoral nerve
Mean age, years	63.4	65.4
Percent male (%)	46.5	46.3
Percent female (%)	53.5	53.7
Mean BMI	31.4	32.2

**Figure 2:**
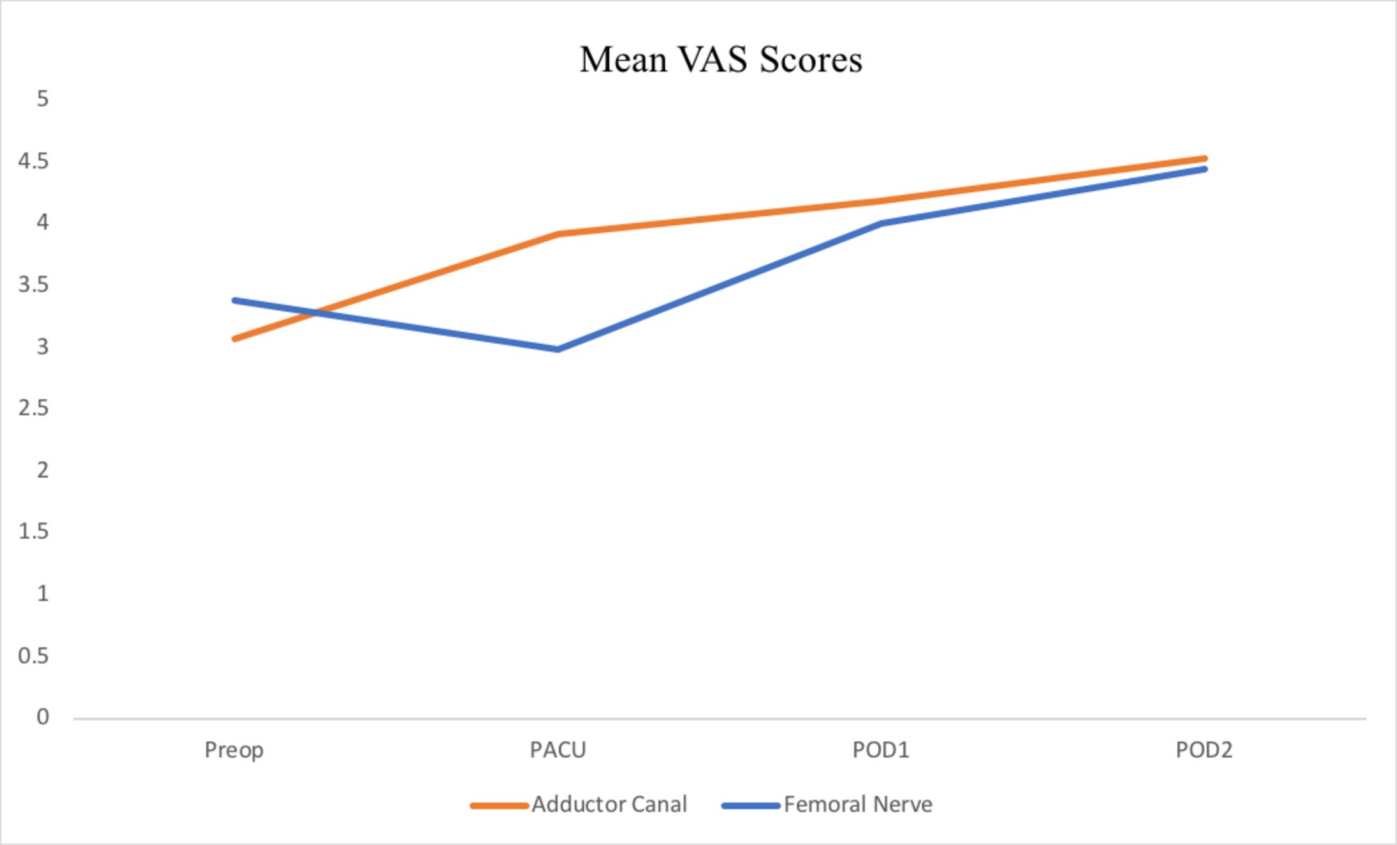
Mean VAS scores for Preop PACU POD1 and POD2 for the ACB and group. VAS, visual analog pain scores; PACU, post-anesthesia care unit; POD, postoperative day; ACB, adductor canal block; FNB, femoral nerve block

Results of comparative outcomes between the AC and FN block cohorts are shown in Table 2. The mean length of stay for participants in the AC and FN groups was 89.66 (±32.24) and 83.78 h (±14.52; p = 0.267), respectively, a difference which was not statistically significant. No significant differences were found between the AC and FN cohorts with respect to VAS pain scores. Scores between AC and FN groups, respectively, were 3.07 and 3.338 preoperatively (p = 0.669), 3.91 and 2.98 in post-anesthesia care unit (PACU) (p = 0.159), 4.18 and 4 on POD 1 (p = 0.736), and 4.53 and 4.44 on POD 2 (p = 0.857) (Figure 2). 

**Table 2:** Mean length of stay, VAS, OME, ambulation distance, satisfaction, and physical therapy participation among all subjects, ACB group only, and FNB group only. VAS, visual analog pain scores; PACU, post-anesthesia care unit; POD, postoperative day; OME, oral morphine equivalent; ACB, adductor canal block; FNB, femoral nerve block

	All subjects	Adductor canal	Femoral nerve	p-value*
Length of stay	86.72 ± 25.04 (n = 84)	89.66 ± 32.24 (n = 43)	83.78 ± 14.52 (n = 41)	0.267
VAS				
Preop	3.22 ± 3.42 (n = 84)	3.07 ± 3.43 (n = 43)	3.38 ± 3.44 (n = 41)	0.669
PACU	3.44 ± 3.14 (n = 84)	3.91 ± 3.19 (n = 43)	2.98 ± 3.05 (n = 41)	0.159
POD1	4.09 ± 2.48 (n = 84)	4.18 ± 2.64 (n = 43)	4 ± 2.35 (n = 41)	0.736
POD2	4.49 ± 2.32 (n = 84)	4.53 ± 2.37 (n = 43)	4.44 ± 2.29 (n = 41)	0.857
OME				
Preop	22.67 ± 8.34 (n = 84)	23 ± 8.52 (n = 43)	22.33 ± 8.23 (n = 41)	0.707
PACU	52.35 ± 33.63 (n = 84)	53.92 ± 34.56 (n = 43)	50.77 ± 32.99 (n = 41)	0.660
POD1	72.16 ± 52.33 (n = 84)	75.54 ± 49.81 (n = 43)	68.78 ± 55.08 (n = 41)	0.543
POD2	49.98 ± 28.13 (n = 84)	55.49 ± 32.3 (n = 43)	44.47 ± 22.26 (n = 41)	0.063
Ambulation				
POD1	59.32 ± 58.05 (n = 84)	70.2 ± 66.94 (n = 43)	48.44 ± 45.76 (n = 41)	0.045
POD2	117.7 ± 88.22 (n = 84)	129.02 ± 92.33 (n = 43)	106.38 ± 83.39 (n = 41)	0.225
Satisfaction	8.98 ± 1.78 (n = 84)	8.71 ± 2.16 (n = 43)	9.24 ± 1.26 (n = 41)	0.156
Participation	8.51 ± 2.01 (n = 84)	8.44 ± 2.2 (n = 43)	8.57 ± 1.81 (n = 41)	0.773

**Figure 3:**
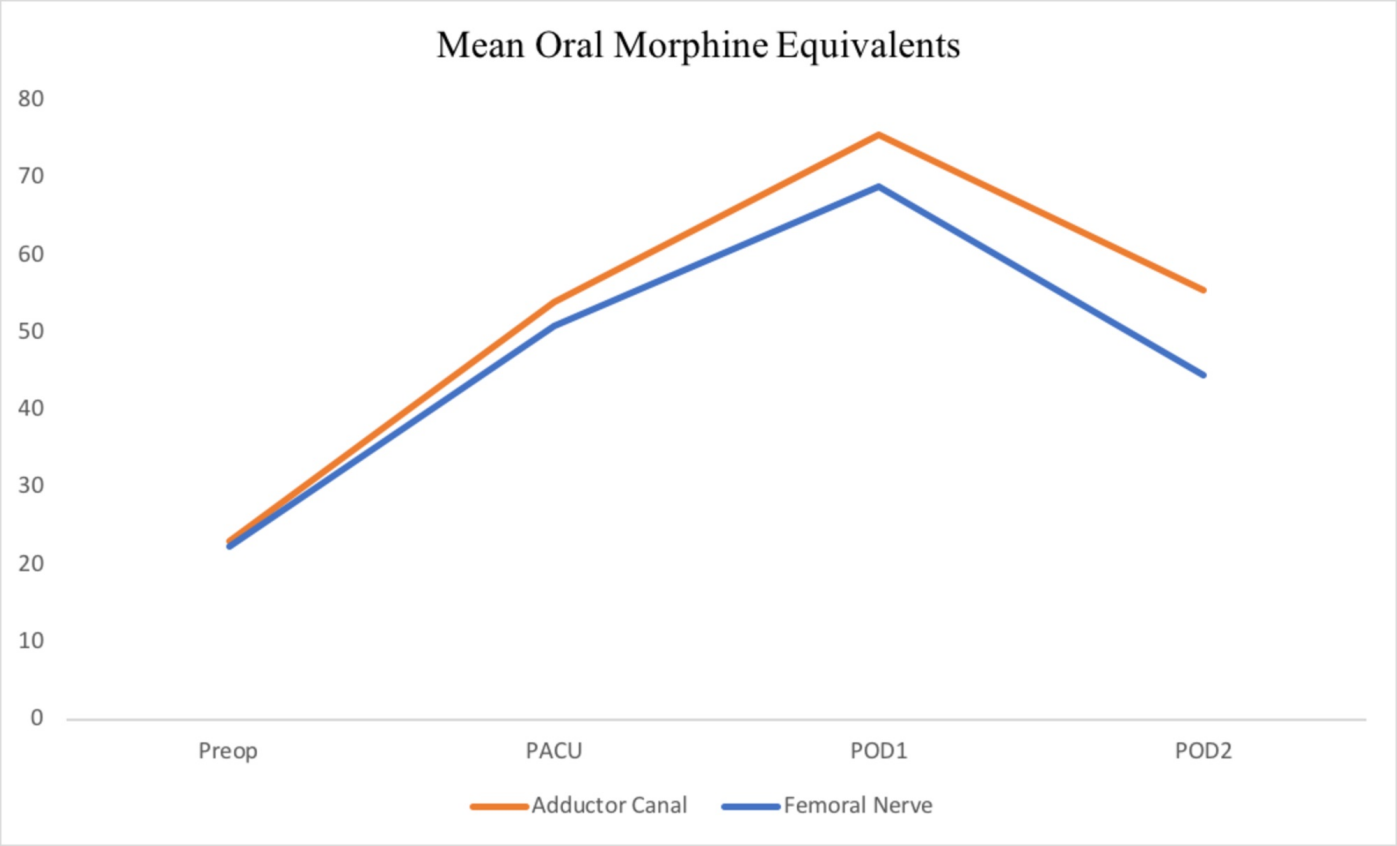
Mean OMEs for Preop PACU POD1 and POD2 for the ACB and FNB group. OME, oral morphine equivalent; PACU, post-anesthesia care unit; POD, postoperative day; ACB, adductor canal block; FNB, femoral nerve block

Similarly, no significant differences in OME were found between the AC and FN groups. OME between the AC and FN groups, respectively, were 8.52 and 8.23 preoperatively (p = 0.707), 53.92 and 50.77 in PACU (p = 0.660), 75.54 and 68.78 on POD 1 (p = 0.543), and 55.49 and 44.47 on POD 2 (p = 0.063) (Figure 3). 

**Figure 4:**
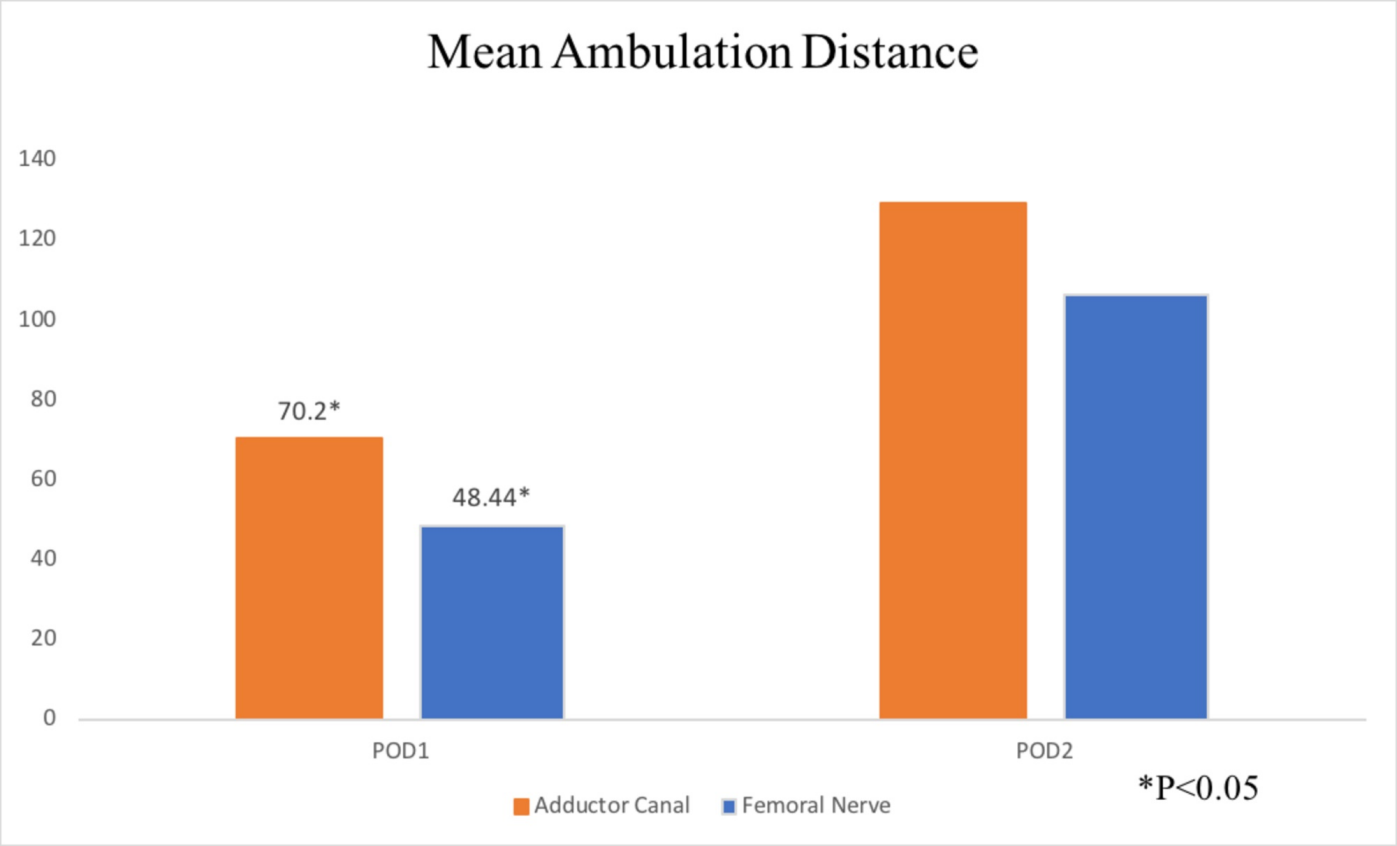
Mean ambulation distance for POD1 and POD2 for the ACB and FNB group. POD, postoperative day; ACB, adductor canal block; FNB, femoral nerve block

Ambulation distance was significantly greater in the AC group (70.2 ft) on POD one (POD 1) than in the FN groups (48.44 ft, p = 0.045). Ambulation distances in the AC and FN groups on POD two (POD 2) were 129.02 and 106.38 ft, respectively, demonstrating no statistically significant difference (p = 0.225) (Figure 4). Patient satisfaction in AC and FN groups, respectively, was 8.71 (±2.16) and 9.24 (±1.26; p = 0.156). Physical therapist-rated participation in physical therapy sessions was 8.44 (±2.2) in the AC group and 8.57 (±1.81) in the FN group, which were also not significantly different (p = 0.773).

## Discussion

Results of this study demonstrated no significant difference in OME used or VAS pain scores among patients undergoing AC block in comparison to patients undergoing FN block, indicating comparable analgesic efficacy between the two techniques. However, patients receiving AC block had significantly greater postoperative ambulation distances on POD 1, demonstrating a transient improvement in motor function among these patients in comparison to patients receiving the FN block.

The most notable and novel finding in our study was the significantly greater ambulatory distance among AC patients on POD 1 in comparison to FN patients, indicating superior early motor function among this cohort. Early ambulation after surgery is important for complication prevention and functional recovery. Labraca et al. emphasized the importance of early rehabilitation following TKA, as those who began physical therapy within 24 h had significantly shorter hospital stay, fewer rehabilitation sessions until discharge, decreased pain, greater range of motion of the joint, improved strength, and higher gait and balance scores in comparison to those who started within 72 h [8]. Chandrasekaran et al. showed that among patients who underwent TKA, those who ambulated on POD 1 had significantly lower incidence of thromboembolic complications; odds of thromboembolic complications decreased significantly with increasing mobilized distance, as well [9]. Therefore, utilizing a local regional block which improves early ambulation in patients may reduce risk of postoperative complications and improve overall recovery.

Mudumbai et al. assessed total postoperative ambulation distances on PODs one and two among TKA patients receiving AC and FN block and found significantly greater distances walked for both days among the AC cohort. However, ambulation distances were recorded retrospectively from physical therapy notes [10]. Shah and Jain compared postoperative ambulation between AC and FN patients using the timed-up-and-go test, 10-m walk test, and ambulation distance at discharge, and found that the AC group had significantly better results across all testing modalities [11]. In contrast, a randomized controlled trial by Jæger et al. demonstrated no significant difference in mobilization ability between AC and FN cohorts following TKA as assessed by the timed-up-and-go test, despite quadriceps strength being significantly higher in the AC group [1]. Although previous studies have compared the impact of AC versus FN regional block on postoperative ambulation, to our knowledge this is the first prospective study to directly assess maximum ambulatory distance in the immediate postoperative period.

The effect of block technique on postoperative strength of the quadriceps muscle has also been previously assessed, and the efficacy of the AC block for preservation of quadriceps strength is well documented in the literature. Seo et al. utilized manual muscle testing (MMT) to directly evaluate quadriceps strength, and results demonstrated significantly greater strength among patients receiving AC block compared to patients receiving FN block [12]. Kim et al. demonstrated similar findings, and also directly assessed quadriceps strength using a dynamometer [4]. Results of a meta-analysis by Li et al. also showed that FN block resulted in decreased quadriceps strength in comparison to those receiving AC block [13]. Our study did not directly assess quadriceps strength as previous studies have consistently demonstrated that AC block preserves quadriceps strength. Our study assessed and compared the functional outcome in the form of ambulation between two groups. From our findings of significantly increased ambulation distance among the AC group on POD 1, findings of previous literature, and understanding of the fundamental role of the quadriceps in ambulation, it can be assumed that quadriceps strength was likely greater among these patients as well, which would improve overall functionality.

In addition to functional recovery, postoperative pain management is also a fundamental principle of postoperative management essential for patient satisfaction and rehabilitation. Multiple studies have previously assessed the analgesic efficacy of the AC block in comparison to the well-established efficacy of the FN block through comparison of pain scores and opioid consumption, and the results of our study are comparable to those of previously published literature. Prospective randomized controlled trials by Shah et al. and Kim et al. demonstrated no statistically significant difference in reported postoperative pain scores between the two groups [4, 11]. Similarly, a retrospective study by Seo et al. compared numeric rating scale (NRS) pain scores between groups postoperatively and found that overall, no significant difference existed between the two analgesic techniques for pain relief in TKA [12]. Of note, transient occurrences of significantly greater pain scores among the FN group compared to the AC group were found at rest on PODs 1, 2, and 3, and with the knee at 45 degrees of flexion on days 1 and 2, which were inconsistent with our findings. However, the transient increases in pain scores in the FN group further support the potential superiority of the AC block for preserving motor function without compromising analgesic efficacy.

Well-controlled pain not only improves patient satisfaction, but also minimizes the need for narcotic pain medication postoperatively, thereby reducing risk of associated side effects including addiction and its potential consequences. Kim et al. demonstrated no significant difference in opioid intake between the FN and AC groups [4]. Similarly, Jæger et al. found comparable total morphine consumption between AC and FN groups after TKA [1]. Rather than assessing total opioid consumption, Shah et al. evaluated the number of patients in AC and FN groups who required injectable rescue tramadol postoperatively; again, no significant difference was observed [11]. The results of our study concur with established literature assessing opioid intake between the two groups. FN block is the standard regional block for pain relief; therefore, established comparable relief from AC block indicates promising outcomes for patients who are subjected to this method. Establishment of an analgesic regimen which preserves motor function while providing sufficient pain relief is necessary for patient comfort and recovery.

Limitations of this study should be taken into consideration. A sample size is relatively small, and not representative of the entire population. The ambulation on POD 2 is trending in favor of ACB, but not clinically significant due to small sample size. There may be other patient-related factors affecting the ability to ambulate after TKA like age, obesity, present history of back pain, osteoarthritis of nonoperated knee. Further subgroup analysis and bigger sample size may be warranted to compare the effects of ACB and FNB on ambulation.

In comparison to FNB, ACB provides similar analgesia and patient satisfaction but allows for earlier and superior ambulation in the immediate postoperative period. ACB is a viable option for adequate pain relief while simultaneously preserving motor function in the early postoperative period, which may ultimately result in improved patient outcomes.

## Conclusions

In comparison to FNB, ACB provides noninferior analgesia and patient satisfaction but allows for superior ambulation only in the immediate (POD 1) postoperative period. ACB is a viable option for adequate pain relief while simultaneously preserving motor function in the early postoperative period, but our study did not find any significant improvement in ambulation beyond 24 h postsurgery. Further studies are warranted to investigate long-term effects of improved ambulation and functional recovery with ACBs.
